# Design of a Small-Scale Multi-Inlet Vortex Mixer for Scalable Nanoparticle Production and Application to the Encapsulation of Biologics by Inverse Flash NanoPrecipitation

**DOI:** 10.1016/j.xphs.2018.05.003

**Published:** 2018-09

**Authors:** Chester E. Markwalter, Robert K. Prud'homme

**Affiliations:** Department of Chemical and Biological Engineering, Princeton University, Princeton, New Jersey 08544

**Keywords:** nanoparticles, mixing, protein delivery, peptide delivery, formulation, particle size, polymeric drug carrier, FNP, Flash NanoPrecipitation, CIJ, confined impinging jets, MIVM, Multi-Inlet Vortex mixer, NP(s), nanoparticle(s), BCP, block copolymer, PS-PEG (PS-*b*-PEG), poly(styrene)-*block*-poly(ethylene glycol), PS, poly(styrene), THF, tetrahydrofuran, Re, Reynolds Number, iFNP, inverse Flash NanoPrecipitation, μMIVM, micro Multi-Inlet Vortex Mixer, CHCl_3_, chloroform, DMSO, dimethyl sulfoxide, PS-b-PAA, poly(styrene)-block-poly(acrylic acid), DLS, dynamic light scattering, PDI, polydispersity index, OVA, ovalbumin, HRP, horseradish peroxidase

## Abstract

Flash NanoPrecipitation is a scalable approach to generate polymeric nanoparticles using rapid micromixing in specially designed geometries such as a confined impinging jets mixer or a Multi-Inlet Vortex Mixer (MIVM). A major limitation of formulation screening using the MIVM is that a single run requires tens of milligrams of the therapeutic. To overcome this, we have developed a scaled-down version of the MIVM, requiring as little as 0.2 mg of therapeutic, for formulation screening. The redesigned mixer can then be attached to pumps for scale-up of the identified formulation. It was shown that Reynolds number allowed accurate scaling between the 2 MIVM designs. The utility of the small-scale MIVM for formulation development was demonstrated through the encapsulation of a number of hydrophilic macromolecules using inverse Flash NanoPrecipitation with target loadings as high as 50% by mass.

## Introduction

Nanoparticles (NPs) have received considerable interest as vehicles for hydrophobic therapeutics and imaging agents, among other applications.^1^, ^2^, ^3^, ^4^, ^5^ Production of polymeric NPs in a reproducible manner is necessary for successful clinical translation, but scale-up is often difficult.^5^, ^6^ Flash NanoPrecipitation (FNP) provides a scalable solution to this challenge through a continuous rapid mixing process, which is carried out in specific geometries.^2^, ^7^ As a continuous process, scale-up entails longer run times rather than implementation in larger vessels.^8^, ^9^, ^10^ The resultant NPs contain a core of the hydrophobic organic active(s) with the surface sterically stabilized by an amphiphilic block copolymer (BCP). This architecture means the percent mass from the active can be large, with therapeutic loadings up to 90%.

**Figure 1:**
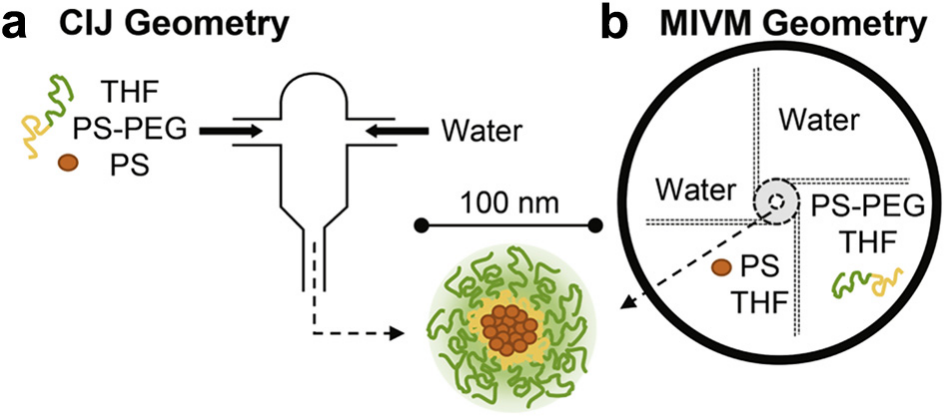
(a) Schematic of the CIJ mixer and example stream compositions for FNP. (b) Schematic of the MIVM mixer and example stream compositions for FNP. The NP structure produced by FNP is displayed schematically and does not reflect the relative core and stabilizer dimensions.

In the original report of FNP, Johnson and Prud'homme^7^ described a confined impinging jets (CIJ) mixer to achieve the necessary rapid mixing. In this process, shown in Figure 1a, one of the inlet streams is an organic solvent containing molecularly dissolved BCP and hydrophobic active. The other stream contains a miscible antisolvent, typically water, to drive precipitation of the active and 1 block of the BCP. The streams are impinged collinearly with equal momentum. The rapid change in solvent quality leads to high supersaturation and initiates nucleation of the hydrophobic active, which grows to form the particle core. Particle growth is uniformly halted by the assembly of the insoluble polymer block onto the particle surface. At sufficient inlet stream velocity, the time scale for mixing is faster than the aggregation time of the active and the BCP. Particles with low polydispersity are formed under these “homogeneous” conditions.^7^, ^9^

The CIJ design requires 2 inlet streams with equal momenta, which limits the supersaturation that can be reached. This limitation is addressed by the Multi-Inlet Vortex Mixer (MIVM) geometry, shown schematically in Figure 1b.^11^ In this 4-inlet design, each stream can be considered to independently contribute to the micromixing in the vortex chamber. Using competitive reactions to characterize the mixing time scale, Liu et al.^11^ found that the MIVM achieved sufficient micromixing at Reynolds number (Re) above 1600. The Re is defined by Equation 1, where V_i_ is the average inlet velocity of the ith stream, ν_i_ is the kinematic viscosity, and D is the chamber diameter (Table S1 in Supplemental Information). The MIVM design allows operation at a solvent ratio other than 50% antisolvent. This ratio and, consequently, the supersaturation can be controlled by varying the stream velocities using programmable syringe pumps. With this design, reactive compounds can be segregated or more than 2 solvent types can be employed.

(1) Re=∑ViνiD

Recently, we adapted FNP for the encapsulation of hydrophilic biologics such as peptides and proteins.^12^, ^13^, ^14^ This process is termed “inverse” Flash NanoPrecipitation (iFNP) for clarity, but it relies on the same rapid micromixing principles described previously. The biologic is dissolved in a hydrophilic organic solvent such as dimethyl sulfoxide (DMSO) along with an amphiphilic BCP containing a polyanionic hydrophilic block. The antisolvent stream is a hydrophobic organic solvent such as dichloromethane. The NPs consist of a hydrophilic core with a corona formed of the hydrophobic polymer block. Ionic cross-linking of the polyanionic core stabilizes the NPs for further processing.^13^ The NPs can then be incorporated into either microparticles for sustained release applications or coated with an additional BCP to produce water dispersible NPs.^14^ Such delivery constructs can be employed to reduce the injection frequency typically required for biologics because of their rapid clearance.^15^ iFNP provides significant improvements over existing fabrication techniques in terms of biologic loading and encapsulation efficiency.^12^, ^14^

From a practical perspective, the high cost of many biologics necessitates minimizing material requirements for formulation development. A hand-held version of the CIJ was developed by Han et al.^16^ and enables a single run using 2.5 mg of therapeutic under typical iFNP conditions. However, it is desirable to use more than 2 inlet streams when formulating proteins by iFNP to limit organic solvent exposure. Therefore, we present the design and processing validation of a scaled-down version of the original MIVM design, which we call a “micro” Multi-Inlet Vortex Mixer (μMIVM).

The design removes MIVM features that contribute to large hold-up volumes and reduces the mixing chamber volume to 0.015 cm^3^ from 0.042 cm^3^ (see Table S1 in Supplemental Information). We demonstrated μMIVM operation with a total hold-up volume of about 300 μL for formulations requiring as little as 0.2 mg of therapeutic. This represents a reduction in material requirements of more than an order of magnitude from the original MIVM design. Alternatively, the μMIVM can be run continually using pumps to enable larger batches. We characterized the μMIVM performance using poly(styrene) (PS) as a hydrophobic core and poly(styrene)-*block*-poly(ethylene glycol) (PS-*b*-PEG) as the BCP because we have previously characterized this system in the CIJ geometry.^17^ The flow rate dependence and stream configuration effects on particle size were evaluated using this formulation. We then demonstrated the utility of the μMIVM for iFNP with 3 different biologic formulations.

## Mixer Design

**Figure 2:**
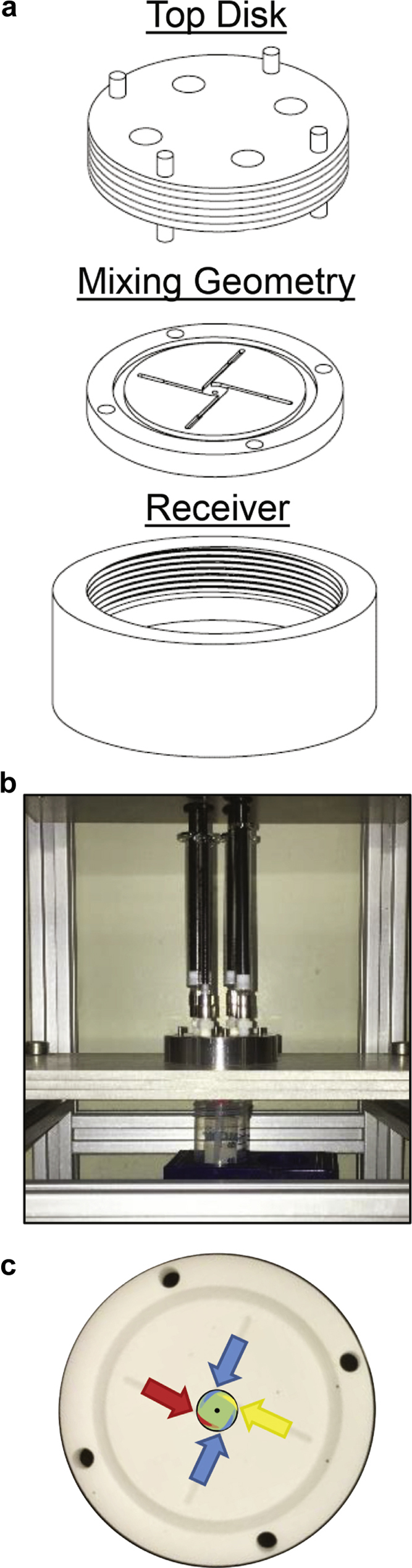
(a) The 3 components of the μMIVM which are assembled together. The syringe adapters on the top disk and the outlet tubing on the receiver are not depicted. (b) The fabricated mixer with 1 mL syringes attached by Luer lock adapters to the top disk and placed in the custom stand. (c) Stream flow schematic in the mixing chamber illustrating the vortex formation in the mixer chamber.^11^

The μMIVM consists of 3 separate components (Fig. 2a). We also constructed a stand (Fig. 2b) which is used to uniformly depress the syringes that hold the inlet streams. A tube or vial is placed beneath the outlet tubing to collect the effluent. Detailed dimensions of the components and the stand design may be found in Supplemental Information Section A.

The top disk is machined from stainless steel for solvent compatibility, and the outer edge is threaded to fit the bottom receiver. It consists of 4 inlets with Luer lock adapters for syringe attachment. They may also be adapted to attach tubing for syringe pump operation at the expense of greater hold-up volume. Around the perimeter, 4 dowels are press fit to project both above and below the disk. These dowels ensure precise alignment of the inlets with the mixing geometry. They also provide points of contact for assembly with a spanner machined to fit the dowel configuration (Fig. S12). This spanner is used to securely tighten the assembly.

The middle piece may be machined from stainless steel or from a solvent-compatible thermoplastic such as Delrin^®^. We designed a separable mixing geometry to enable easy exchange of different designs and for thorough cleaning. The inlet channels are smaller than the original MIVM, and the design has been modified to minimize hold-up, which is reduced to volumes of about 17 μL for a single channel. Hold-up volume for the fully assembled mixer is about 300 μL. The mixing chamber diameter (D) was scaled with the inlet channel dimensions (width, w; height, h) such that residence time in the chamber was constant for a given inlet velocity. This scaling equation is supplied in Supplemental Information Section A, Equation 1. The inlets to this channel create the vortex motion required for rapid micromixing, shown schematically in Figure 1c.^11^ The outlet diameter was scaled linearly with the chamber diameter. A grove for an O-ring encompasses the mixing geometry.

The bottom receiver may be machined from brass or stainless steel because it does not contact the process stream. If stainless steel is used, anti-seize is applied to the threading to avoid galling when assembling the top disk. A fitting for outlet tubing abuts to the middle piece, aligned with the outlet of the mixing chamber to form a liquid-tight seal.

For most applications, we attach gas-tight syringes (Hamilton Company, Reno, NV) to the top disk fittings as shown in Figure S13. The relative flow rates of the streams are controlled by employing syringes of different diameters. As shown in Figure S13, the syringes were modified with set screws so the final height of all syringe sizes was consistent at 11.2 cm. A custom-built stand to hold the mixer and depress the syringes simultaneously is shown in Figure 2b and Figure S9. Shaft clamps are set at the final travel limit to avoid damage to the glass syringes. The push plate, attached to linear bearings mounted on parallel shafts, allows rapid motion and even depression of the syringes. The stream mixing behavior is shown schematically in Figure 2c and has been described in the literature.^11^, ^18^

## Materials and Methods

### Materials

The top disk and bottom receiver were manufactured from stainless steel, and the mixing geometry was made of Delrin^®^ (McMaster-Carr, Robbinsville, NJ). The O-ring was FKM, 35.5 mm × 1.5 mm (C.E. Conover, Bensalem, PA). The syringe fittings were ¼″ luer fittings (P-604; Idex, Oak Harbor, WA). The outlet fitting was ¼″ VacuTight (P-942x; Idex). Albumin from chicken egg white (ovalbumin [OVA], lyophilized powder, >98%), horseradish peroxidase (HRP) (highly stabilized, essentially salt free), and zinc nitrate (Zn(NO_3_)_2_) hexahydrate (reagent grade, 98%) were purchased from Sigma-Aldrich (St. Louis, MO). Dextran T20 was purchased from Pharmacia Fine Chemicals (Uppsala, Sweden). Optima^®^ chloroform (CHCl_3_), HPLC grade tetrahydrofuran (THF, unstabilized), and HPLC grade DMSO were purchased from Fisher Scientific. Poly(styrene)-block-poly(acrylic acid) (PS-*b*-PAA, 4.8k-*b*-5k, polydispersity index [PDI] 1.4), PS (1.8k, PDI 1.08), and PS-*b*-PEG (1.6k-*b-*5k, PDI 1.10) were purchased from Polymer Source (Dorval, Quebec). All reagents were used as received. Deionized water was treated to a resistivity of 17.8 mΩ-cm or greater (NANOpure Diamond; Barnstead International, Dubuque, IA).

### Typical FNP Protocol With the μMIVM

A typical protocol and required parts lists are detailed in Supplemental Information Section B. In brief, the μMIVM is first assembled so that all fittings are snug, and the 3 pieces are tightly compressed using the spanner wrench. The 4 inlet stream compositions are prepared and loaded at the desired volume into syringes. The collection vial (with additional diluent, if desired) is positioned below the mixer. After placing the syringe into their fittings, the push plate is then manually brought carefully to rest at the plunger height. NPs are formed by steadily and rapidly depressing the plate to empty the syringes through the mixer.

### Comparison With the MIVM

Identical NP formulations were prepared at different mixing rates using both the large-scale MIVM as well as the μMIVM to determine the effect of the mixer dimensions on the final NP size distribution. In essence, this tested whether the Re (Eq. 1) is the proper dimensionless group for scaling. PS, the core component, was dissolved in THF at 5 mg/mL. Separately, the stabilizer PS-*b*-PEG was dissolved in THF at 5 mg/mL. For trials with the μMIVM in the stand, 1 mL of each solution was loaded into plastic syringes and placed in 2 adjacent positions. Syringes containing 1 mL of water each were placed in the remaining 2 positions. A collection vial containing 16 mL of water was placed beneath the stand. A timer was used to monitor the speed the plungers were depressed, with an estimated uncertainty of 0.3 s. For pump trials with the MIVM and μMIVM, 100 mL syringes were loaded with each of the 4 inlet streams and attached such that the THF streams were adjacent to each other. The syringe pumps were set to the predetermined volumetric flow rates. After a 5-10 mL equilibration volume at the new flow rate, a fresh vial was used to collect the outlet stream. A known volume of this sample was then diluted with water to the same final composition as the μMIVM formulation.

Particle size was characterized by dynamic light scattering (DLS) using a Zetasizer Nano ZS (Malvern, Worcestershire, UK) at 25°C by diluting each sample 10-fold with water. Size distributions were determined from a CONTIN analysis implemented by the Zetasizer software. The PDI is obtained from the Taylor series expansion of the autocorrelation function, which is implemented by the Zetasizer software. A ratio of the second to the first moment is defined as the PDI. Values of 0.1 are generally obtained for monodisperse particles.^19^, ^20^ The DLS analysis for similar formulations has been validated by comparison to TEM images.^21^ The mixer Re was determined from the mixer dimensions (Supplemental Information Section C) and the volumetric flow rate for each stream using Equation 1.

### Stream Orientation Effects

**Figure 3:**
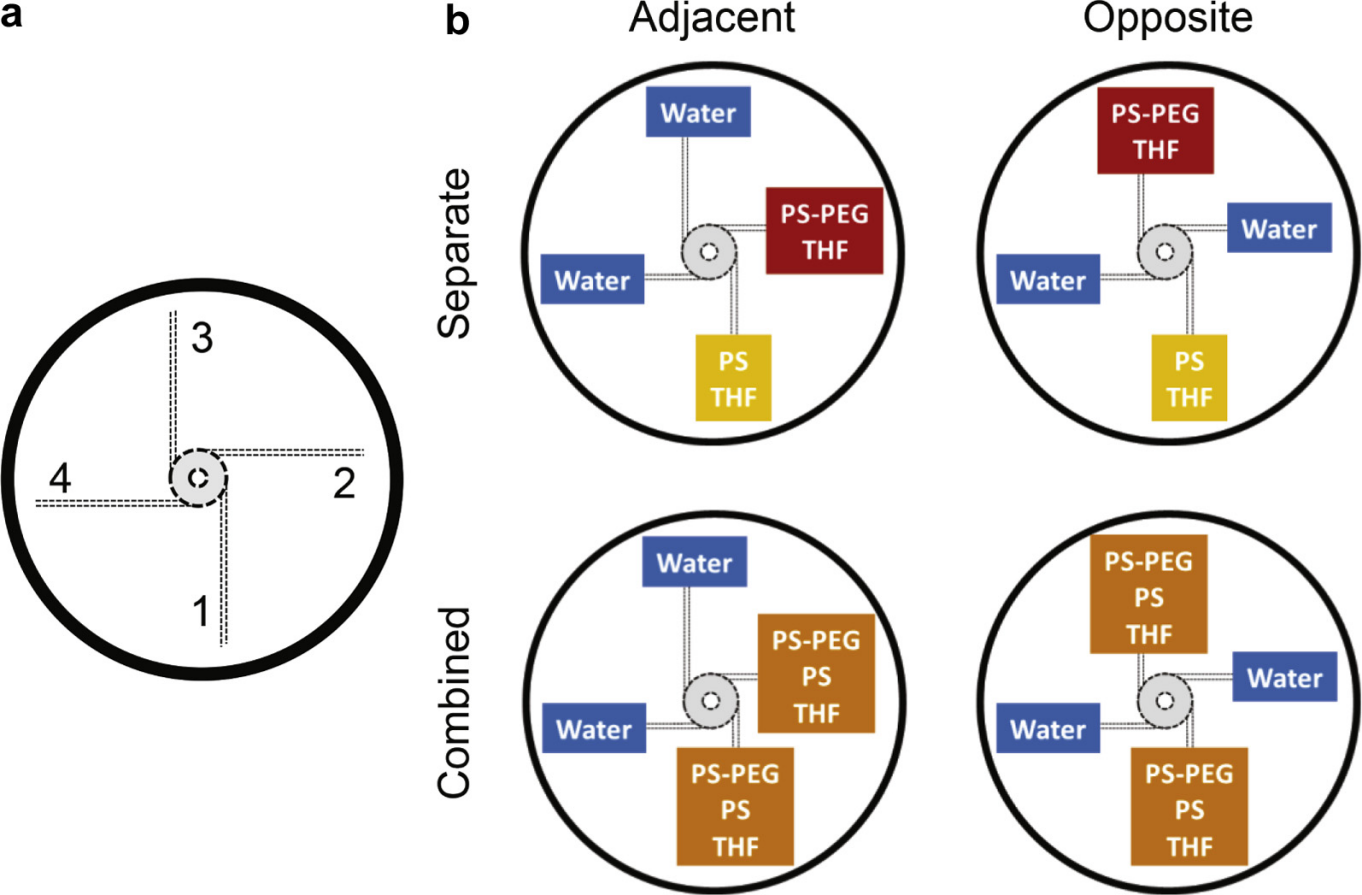
(a) Stream numbering scheme used in Table S4 to depict relative orientation. (b) Schematic of the stream orientations studied using PS/PS-PEG NPs. The column labels indicate whether the organic solvent streams were located adjacent or opposite each other. The row labels indicate whether the core and stabilizer were separated or combined in a single stream.

An initial question is whether the choice of stream orientation or the component distribution affects NP formation and size. Computational fluid dynamics showed that the mixing in the MIVM occurs at the outlet of the chamber, with the 4 streams spiraling with increasing velocity toward the exit.^11^ The orientation effect was evaluated in the μMIVM with the same formulation conditions described previously. The generic orientation labeling scheme is shown in Figure 3a, and specific conditions are displayed in Figure 3b. For formulations where PS and PS-*b*-PEG were combined, 2 syringes were loaded with 1 mL of a solution containing 5 mg/ml PS and 5 mg/ml PS-*b*-PEG and placed at the desired location, either adjacent or opposite each other. NPs were produced and characterized as previously described.

### Dextran iFNP Formulation

Dextran was used as a model hydrophilic active for encapsulation by iFNP. It was dissolved along with PS-*b*-PAA in a solution of 10% water in DMSO at 5 mg/mL each. Then, 200 μL of this solution was loaded into a 500 μL gas tight syringe and attached to the μMIVM. This corresponds to 1 mg of the biologic in a formulation run. Three 2.5 mL gas-tight syringes containing 1 mL of CHCl_3_ were fitted to the remaining inlet positions as shown in Table S4. Rapid depression with the push plate afforded NPs with 50% target loading of dextran, which were characterized by DLS with CHCl_3_, the antisolvent, as a diluent. The DLS analysis of similar formulations has been corroborated by TEM measurements.^14^ This formulation was prepared in triplicate to assess μMIVM reproducibility.

### Ovalbumin iFNP Formulation

To demonstrate how the μMIVM enables iFNP formulations that were not possible with the 2 inlet CIJ mixer, ovalbumin (OVA) was encapsulated at 50% target biologic loading. Rapid precipitation of OVA required separate THF and CHCl_3_ antisolvent streams as well as higher anti-solvent volume ratios than permitted by the CIJ. Each formulation required 2.5 mg of protein, which was dissolved in water at 50 mg/mL and was then diluted with DMSO to afford a 5 mg/mL solution in DMSO containing 10 vol% water. Then, 500 μL of this solution was loaded into a syringe and fitted to the μMIVM. A solution of PS-*b*-PAA in THF at 5 mg/mL was prepared, and 500 μL was loaded onto the μMIVM. The third syringe contained 500 μL of CHCl_3_ with 10 vol% methanol and 6.7 mg/mL zinc nitrate hexahydrate, Zn(NO_3_)_2_, as a cross-linker. This concentration corresponded to a 1:1 charge ratio of the Zn^2+^ cation to the acid groups on poly(acrylic acid). The fourth syringe contained 500 μL of CHCl_3_. This formulation is summarized in Table S4. NPs were formed using the μMIVM and were collected in a tube containing 4 mL of CHCl_3_. The particle size was characterized by DLS using CHCl_3_, the antisolvent, as a diluent.

### Horseradish Peroxidase iFNP Formulation

A formulation of horseradish peroxidase (HRP) requiring 0.2 mg per run was prepared at 30% target loading using the μMIVM. The first inlet stream was 200 μL of a 10 vol% water in THF solution containing 1 mg/mL HRP and 2 mg/ml PS-*b*-PAA. HRP was first dissolved in water followed by dilution with THF, which did not result in precipitate formation. The second inlet stream contained 1 mL of CHCl_3_ with 10 vol% methanol and 0.4 mg/mL Zn(NO_3_)_2_ to afford a 1:1 charge ratio of the Zn^2+^ cross-linker with the poly(acrylic acid) acid groups. The remaining 2 inlet streams contained 1 mL of CHCl_3_. Table S4 contains a summary of the formulation. The NP was produced using the μMIVM and collected in a tube containing no additional diluent. The particle size was characterized as described for dextran NPs.

## Results and Discussion

### Comparison of Mixer Designs

**Figure 4:**
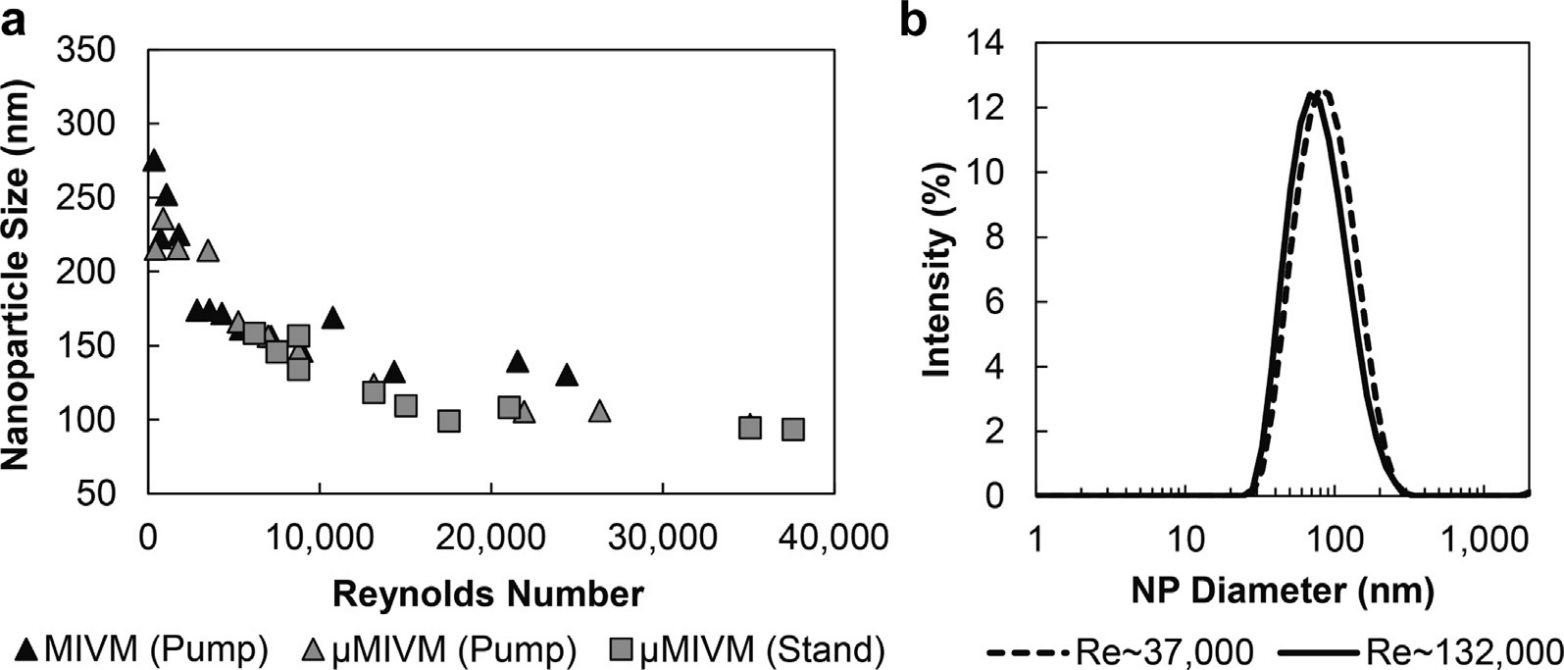
(a) NP size dependence on mixer Re, as defined in Equation 1, for polymeric NPs consisting of a PS core and stabilized by PS-b-PEG, in the 2 mixer designs. The μMIVM was tested using syringe pumps and the custom stand to demonstrate agreement across production scales. The graph shows good agreement over the range of overlapping conditions, indicating that Re is the correct basis for mixer size scaling. (b) PS/PS-PEG NP size distributions produced at Reynolds numbers that correspond to low PDI (Re∼37,000) and high PDI (Re∼132,000). The PDI values were 0.16 and 0.22, respectively. A “dust” population with particle size above 5000 nm is not shown for clarity. Figure S18 illustrates that this population is a measurement artifact.

To test the function of the μMIVM, PS-*b*-PEG–stabilized NPs containing PS homopolymer in the particle core were produced using a syringe pump at different flow rates for comparison with those produced using the MIVM. The μMIVM was also tested using a custom stand to drive syringes attached directly to the mixer (Fig. 2b) to validate performance during small-scale formulation screening. The larger size and flow rates for the MIVM required operation using pumps. NP size was evaluated by DLS at different flow rates, which were converted to mixer Re (Eq. 1) and are reported in Table S2 and Figure 4a for the 3 configurations tested. The expected transition from large particles in the poor mixing region at low Re to a weak dependence at Re∼2000-4000 can be observed in Figure 4a, in good agreement with Liu et al.^11^ This transition appears to occur at a slightly higher Re value for the μMIVM. This may reflect an error in mixer dimension measurement but is not consequential because typical operation is at higher Re values. The overlapping curves for the 2 mixers indicate no functional differences between the 2 designs. The excellent agreement between stand- and pump-driven flow configurations for the μMIVM indicates that small-scale operation scales directly to larger batch sizes. This overlap also indicates that the start-up volume to achieve steady-state is negligible and does not impact NP size for small batches. The primary source of uncertainty in Re for each data point is the volumetric flow rates. For the pump-driven flow, this is minimal as a result of the computer-controlled syringe pump. For the stand-driven flow, the uncertainty in the time to depress the syringes was about 0.3 s with depression times ranging from 0.6 s to 8.5 s.

Re values up to 132,000 are experimentally accessible with the μMIVM with stand-driven operation (Fig. S14). This is significantly higher than for the MIVM, where the limit is Re∼25,000. This difference derives from the upper limit on the flow rate imposed by the syringe pumps used with the MIVM. However, the agreement in regions of Re overlap shows that the MIVM would match the μMIVM particle size at Re = 132,000 if larger metering pumps were used to achieve the flow rate of 360 mL/min corresponding to this Re value. The direct translation from small scale formulation development with the μMIVM to commercial scale production with the MIVM is demonstrated by these data.

We observed a slight upward trend in the polydispersity of particles produced in the μMIVM beginning around Re values above 30,000 or a calculated chamber residence time of 10 ms (Figs. S15 and S16). Modeling of experimental data indicates the assembly time for a 100 nm particle is about 18 ms.^17^ Delaying exposure to the water quench with a 5-fold longer outlet tubing to ensure complete NP assembly did not lead to decreased PDI. With regular tubing, the PDI was 0.24 ± 0.02 while long tubing gave a PDI of 0.23 ± 0.05. These results suggest that, above Re of around 30,000, particles exit the chamber before assembly is complete and experience shear-induced aggregation in the outlet tubing. Representative NP size distributions produced at Re of 37,000 (near the PDI minima) and 132,000 (the highest achieved Re value) are depicted in Figure 4b. The distributions illustrate that the observed PDI trend represents a minor change in the sample polydispersity.

### Impact of Stream Orientation

The 4 inlet streams of the MIVM and μMIVM add an additional factor that must be studied when developing a formulation: the effect of stream orientation on particle size. Each formulation will have its own constraints imposed by solubility and reactivity. Solutions containing particle components can be placed in channels that enter the mixing chamber at adjacent positions or at opposite positions. To demonstrate the effects of stream position on particle size in the μMIVM, PS/PS-PEG NPs were produced using the 4 orientations shown in Figure 3b. The particles were generated in triplicates at Re values of 90,000.

**Figure 5:**
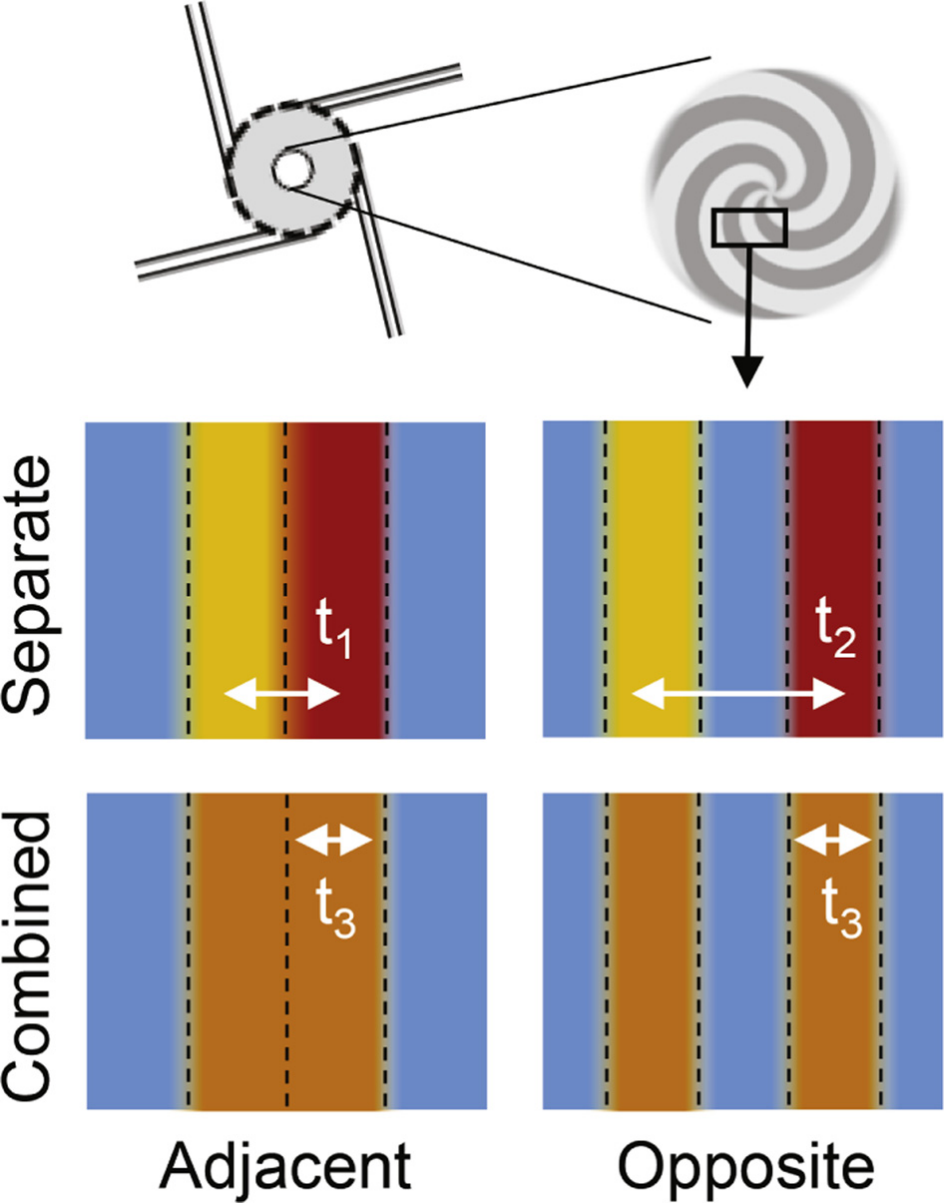
Stream striations (lamellae) form in the mixer on the shortest length scales. Particle assembly is halted by the assembly of BCP stabilizer onto the growing core surface. The largest particles are expected to form for the stream orientation with the widest segregation between the lamellae containing the BCP and the core material. This corresponds to a diffusion time, t, noted on the figure. The diffusion time ranking (t_3_ < t_1_ < t_2_) matches the particle size order (60 nm < 70 nm < 91 nm) in the corresponding experimental configurations. Striation colors match the Figure 3 schematic.

The resulting particle size and standard deviation across triplicate runs are summarized in Table S5. The results match intuitive explanations for mixing behavior in the μMIVM. The smallest particle size, 55-60 nm, was obtained when the components were combined in the same THF stream, regardless of orientation. The largest particles (91 nm) are produced when components are separated and in opposite inlet streams. It should be noted that, even under these conditions, the particles retain low polydispersity (PDI of 0.18), indicative of the uniform assembly expected for the FNP process. Figure 5 depicts the striations or lamellae that develop at the shortest mixing length scales in the chamber through turbulent energy dissipation.^9^ Since particle growth is halted by BCP assembly, diffusion time (t_1_, t_2_, or t_3_ in Fig. 5) dictates particle size. Larger particles are generated in configurations where the distance between striations containing the BCP and the core material are greater.^10^ It should also be noted that the high process reproducibility indicated by the low standard deviation across triplicate preparations of each formulation is an important aspect of successful clinical translation of a formulation.

### Reduced Material Formulations of Biologics in the μMIVM

The primary motivation for development of the μMIVM was to enable formulation development with minimal sample mass. This might be achieved by reducing inlet stream volume in the CIJ, where the lower limit is set by the hold-up volume. As stream volume decreases, the hold-up volume leads to greater processing losses. Furthermore, the requirement for equal momentum means that the antisolvent volume must be reduced as well. A 0.2-mL inlet in the CIJ would result in hold-up losses of around 75%. In the μMIVM, the solvent stream can be reduced to this volume, whereas the antisolvent volume is unmodified. From a practical perspective, the lower volume limitation is about 100 μL because the channel hold-up of 17 μL leads to losses of 20% at this volume. To demonstrate the low volume capability of the μMIVM, we prepared in triplicate a representative formulation which consists of a model hydrophilic macromolecule, dextran, stabilized by PS-*b*-PAA at 50% loading. The formulation in a CIJ requires 2.5 mg of biologic per run.^12^ Modified for low volume in the μMIVM, it requires 1 mg per run. The summary of the stream compositions used in the μMIVM may be found in Table S4, and the stream orientation corresponding to the stream numbers can be referenced against Figure 3a.

**Figure 6:**
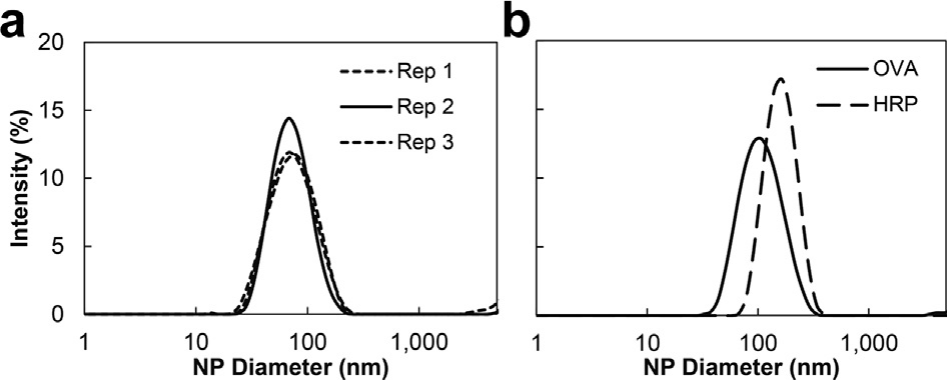
Particle size distribution for (a) triplicate dextran NPs and (b) OVA and HRP NPs prepared by iFNP using the μMIVM. The lower intensity values in (a) for Rep 1 and Rep 3 reflect a larger “dust” population detected by DLS, which can be partially seen in the data above 1000 nm. This figure illustrates the high reproducibility and narrow distributions produced in the μMIVM.

The particle size distributions for the triplicate runs are shown in Figure 6a. There is excellent agreement between the replicate preparations of the same formulation. The particle size was 78 ± 3 nm with a polydispersity of 0.24 ± 0.03. The size was smaller than NPs from the CIJ mixer (147 ± 3 nm).^12^ The 1:15 solvent:antisolvent mixing ratio in the μMIVM results in higher supersaturation relative to the 1:1 ratio in the CIJ. Higher supersaturation drives higher nucleation rates, which produces smaller particles.^2^

The expanded number of possible inlet streams enables formulations with the MIVM that would not be achievable with the CIJ. To demonstrate this, we formulated OVA, a common model vaccine antigen, at a target loading of 50% by mass using the μMIVM. The 2 stream configuration in the CIJ was not able to produce NPs because the biologic did not precipitate at the 1:1 solvent:antisolvent mixing ratio. The μMIVM formulation, requiring 2.5 mg of OVA per run, is summarized in Table S4, and the resulting particle size distributions are shown in Figure 6b. The formulations afforded 115 nm particles with a PDI value 0.17. Without a BCP stabilizer, large aggregates are produced. The μMIVM permitted the addition of a THF stream and reduction of the DMSO content in the mixing chamber, which were necessary to achieve rapid OVA precipitation and encapsulation by iFNP.

The use of DMSO to simultaneously dissolve the biologic and the BCP can result in protein denaturation and loss of activity.^22^, ^23^, ^24^, ^25^, ^26^ The problem extends to other organic solvents and remains a barrier in the biologics delivery field.^27^, ^28^ A separate, low-volume aqueous stream for the biologic to reduce solvent exposure is therefore desirable and has been enabled by the multiple inlets of the μMIVM. However, higher water content increases NP size, resulting in a trade-off between using a separate aqueous stream and producing target particle size distributions.^13^ For some proteins, miscible solvents can be added to reduce water content without detriment to protein activity.^23^, ^29^, ^30^ We demonstrated this approach with acetylated HRP, which we have observed to retain residual activity in THF/water mixtures even up to 90 vol% THF (unpublished data). HRP NPs were prepared in the μMIVM at a target 30% loading using 200 μL of a stream containing the enzyme and the BCP. The formulation, requiring only 0.2 mg of biologic per run, resulted in a particle size of 167 nm and PDI of 0.16, shown in Figure 6b. Notably, repeating the experiment with no stabilizing BCP afforded large aggregates in CHCl_3_. This HRP formulation combined a number of techniques to lower biologic mass requirements enabled by the μMIVM. The first was reducing the biologic stream volume to 200 μL. The second was modulating the solubility of the biologic and reducing water input to the iFNP process through the use of THF. The combined result is a formulation requiring only 0.2 mg of biologic per run.

## Conclusion

A redesigned, small-scale Multi-Inlet Vortex Mixer (μMIVM) has been shown to be functionally equivalent to the larger original design. Size control as a function of mixing has been characterized and found to be only weakly dependent on mixing rate for Re above 2000-4000. The Re based on inlet jet velocity was shown to accurately predict flow rate scale-up to the MIVM geometry. The μMIVM attains Re values as high as 132,000. The hand operation of the μMIVM allows rapid formulation screening while permitting easy transition to commercial (kilogram) scale using the MIVM. The μMIVM design is actively being implemented at a contract research organization for preclinical and clinical development of a hydrophobic drug candidate.

We have reported a number of highly loaded formulations of biologics using the iFNP process and the newly designed μMIVM. The smaller scale enables NP formulation screening in a resource-sparing manner, using as little as 0.2 mg of biologic. The iFNP biologic formulations reported here are dispersed in an organic solvent. There are additional processing steps for the formation of microparticles or water-dispersible NPs for *in vivo* drug delivery, which have been reported elsewhere.^14^ A primary focus of our ongoing work is to develop iFNP processing schemes that maintain secondary structure and activity for protein therapeutics. The μMIVM is central to this effort because of its low material usage and ability to segregate proteins from denaturing solvents.
