# Supplementary material for: Design of a Small-Scale Multi-Inlet Vortex Mixer for Scalable Nanoparticle Production and Application to the Encapsulation of Biologics by Inverse Flash NanoPrecipitation
Source: J Pharm Sci. 2018 Sep;107(9):2465–71. doi: 10.1016/j.xphs.2018.05.003 (PMC6095068; doi:10.1016/j.xphs.2018.05.003)
Supplement: Supplemental Information [file mmc1.docx]

**Supplementary Information for:**

**Design of a Small-Scale Multi-Inlet Vortex Mixer for Scalable Nanoparticle Production and Application to the Encapsulation of Biologics by Inverse Flash NanoPrecipitation**

Chester E. Markwalter, Robert K. Prud’homme

1. *Mixer Dimensions and Stand Design Notes*

CAD files or assistance in fabrication of a μMIVM are available upon contacting the authors. Dimensions (in inches) are captured in the following figures which indicate the mixer and the stand design implemented for this work.





Figure S1: Dimensions of the stainless steel “top disk”. Dowels are not included explicitly in this image. They should not project more than the thickness of the Delrin piece. The threading on the outer edge is not shown explicitly. The ¼” machine threading can be modified to fit any fitting/adapter that is desired for the application. This sizing will work for the fittings specified below.

*

*

Figure S2: Middle “mixing geometry” disk with precise dimensions. Fit with the top disk are crucial. This piece can also be made from stainless steel though the machining must be precise and we recommend polishing to avoid channels of liquid forming along the surface, regardless of material choice.

Scaling of the chamber diameter (D_c_) was relative to the channel width according to:

$\frac{D_{C1}^{2}}{w_{1}}=\frac{D_{C2}^{2}}{w_{2}}$ Eqn. S1

Where 1 refers to the MIVM dimensions and 2 refers to the μMIVM dimensions.

*

*

Figure S3: Receiver/base which is machined to fit the top disk.

The stand design is highly flexible and can be modified to fit particular needs or design preferences. The following images convey some key points from the custom design we implemented. Part numbers from McMaster-Carr are in the following section. The structural components are ITEM profiles (extruded aluminum). This stand is necessary for small scale formulation screening because it permits the four syringes to be depressed simultaneously. Alternatively, syringe pumps can be attached directly for more precise control of the flow rates, though this results in long tubing lines that contribute to high hold-up volumes. We recommend a brief “start-up” time where the outlet is collected in a waste container if using syringe pumps.


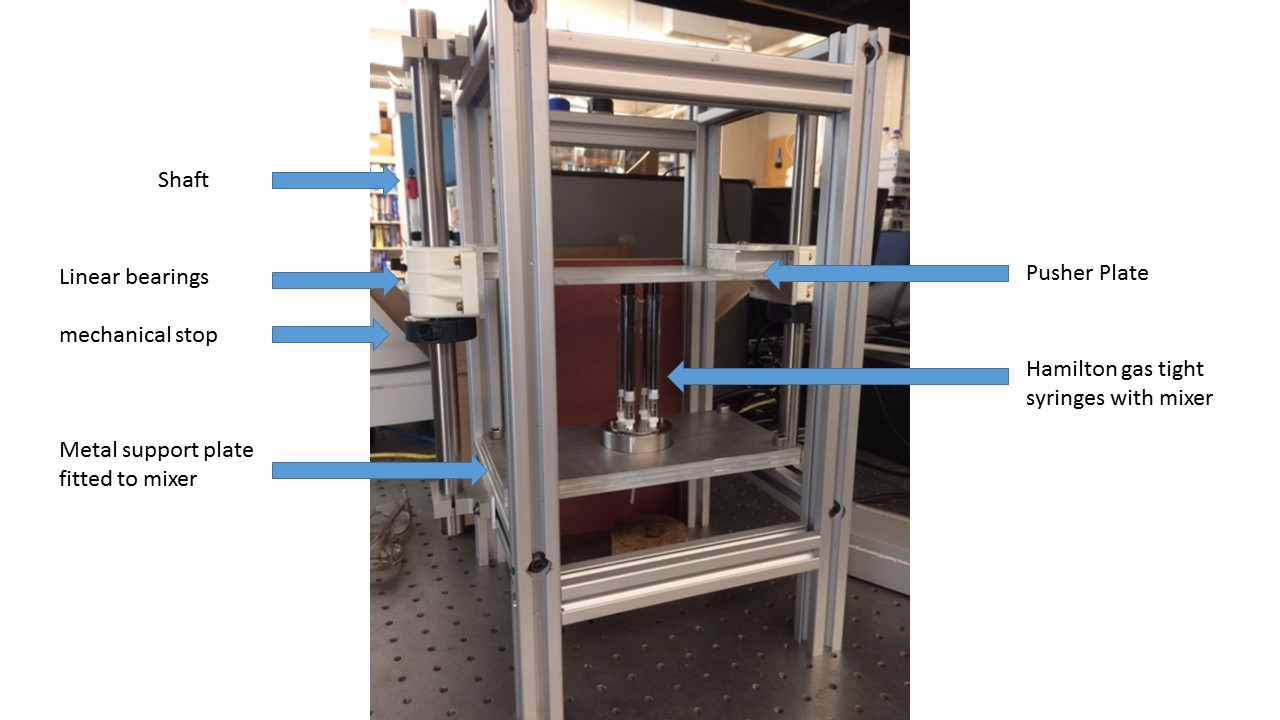


Figure S4: Labeled image of assembled mixer placed in the stand


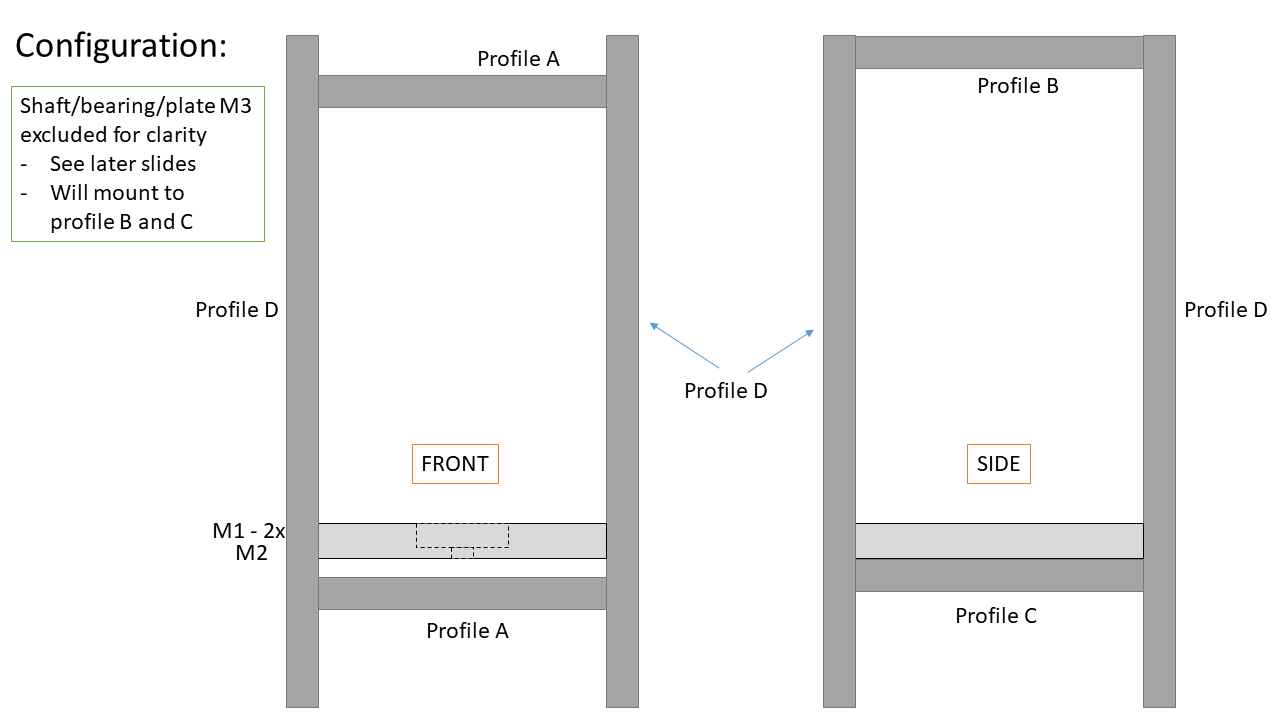


Figure S5: Stand design terminology used in the remaining figures.


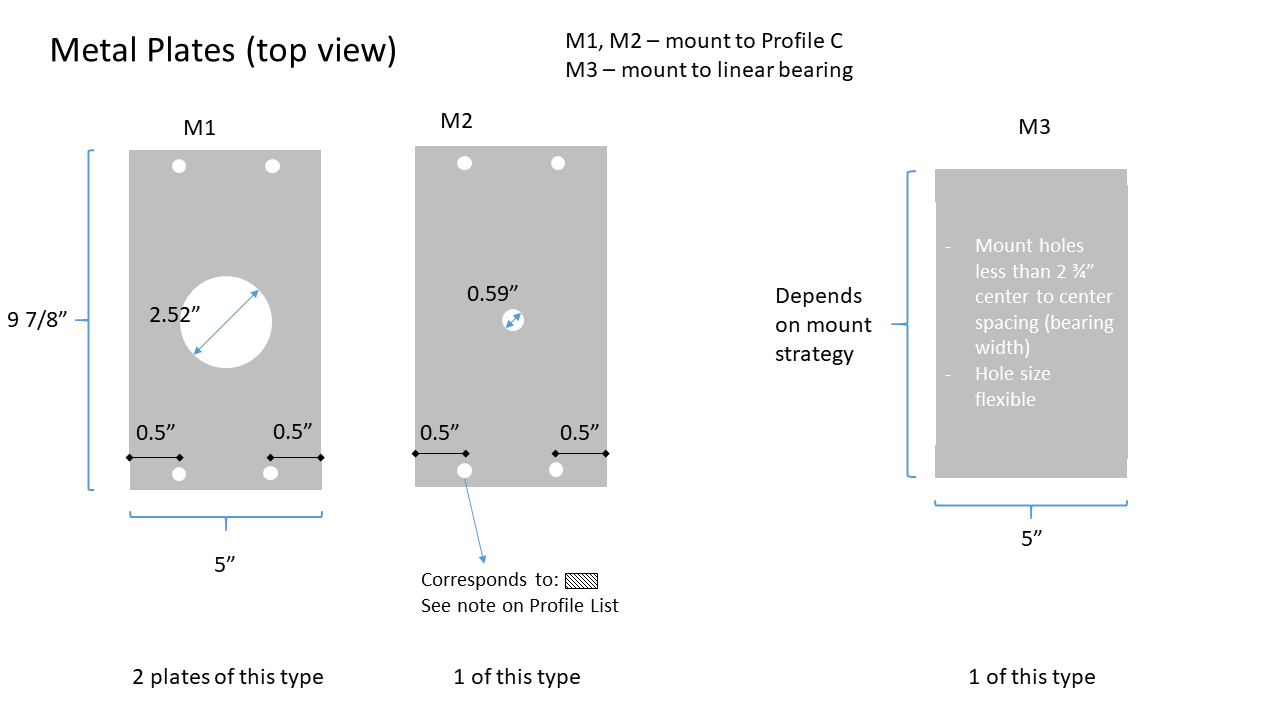


Figure S6: Aluminum plate dimensions used to support the mixer and as the push plate. M1 and M2 are stacked together and attached to the aluminum supports as the place to set the mixer. The 0.59” hole permits the outlet tubing fitting to extend below the place. M3 is the pusher plate and should be mounted as noted below.


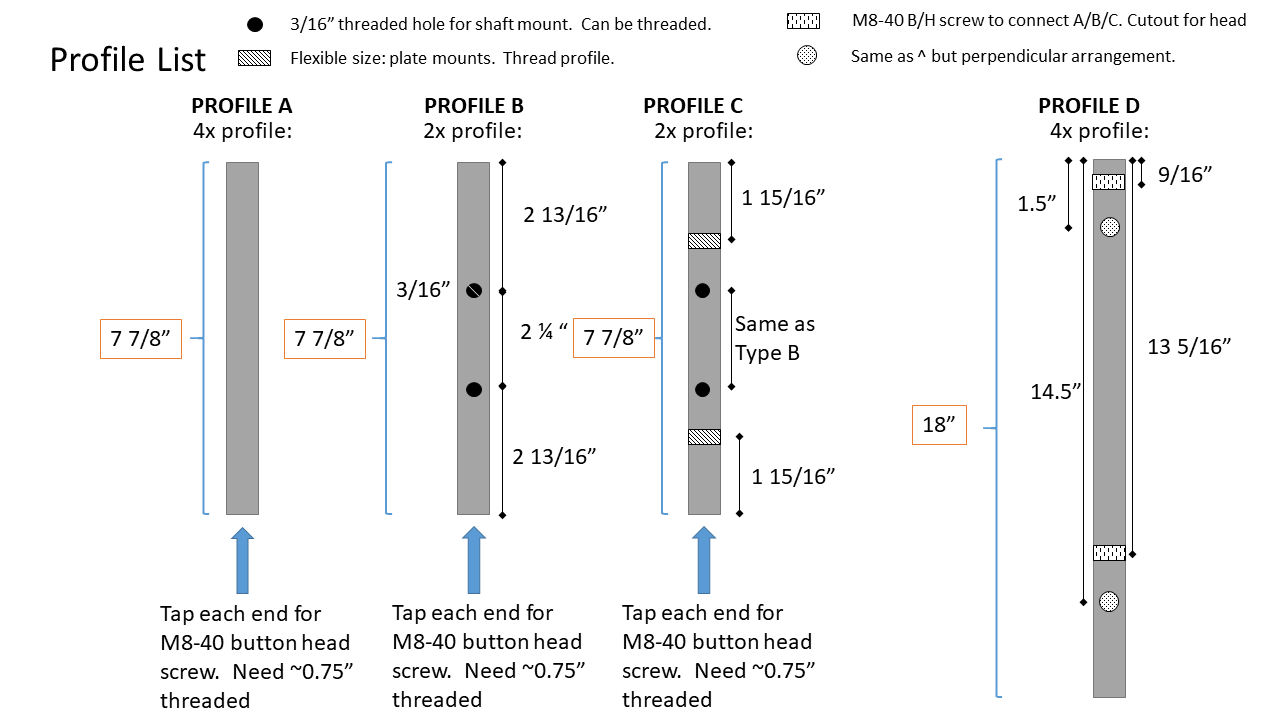


Figure S7: The ITEM profile modifications required for structural assembly.


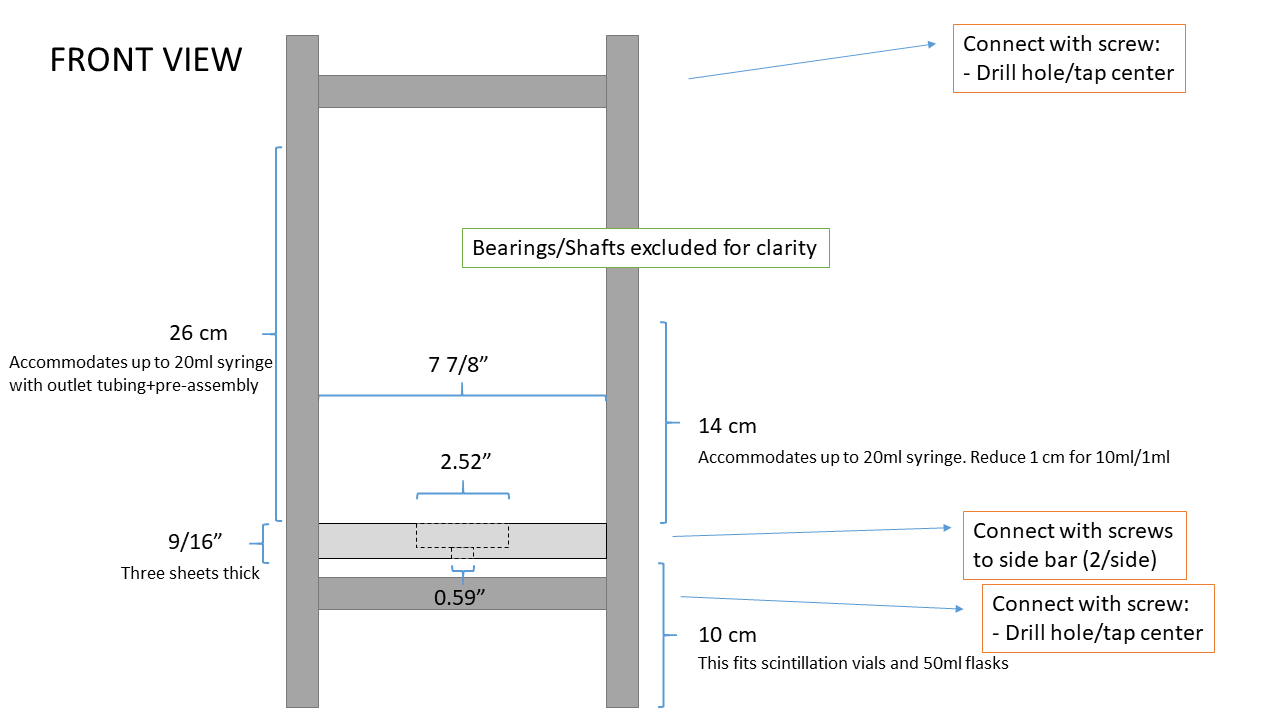


Figure S8: Dimensions of the assembled stand with pertinent notes.


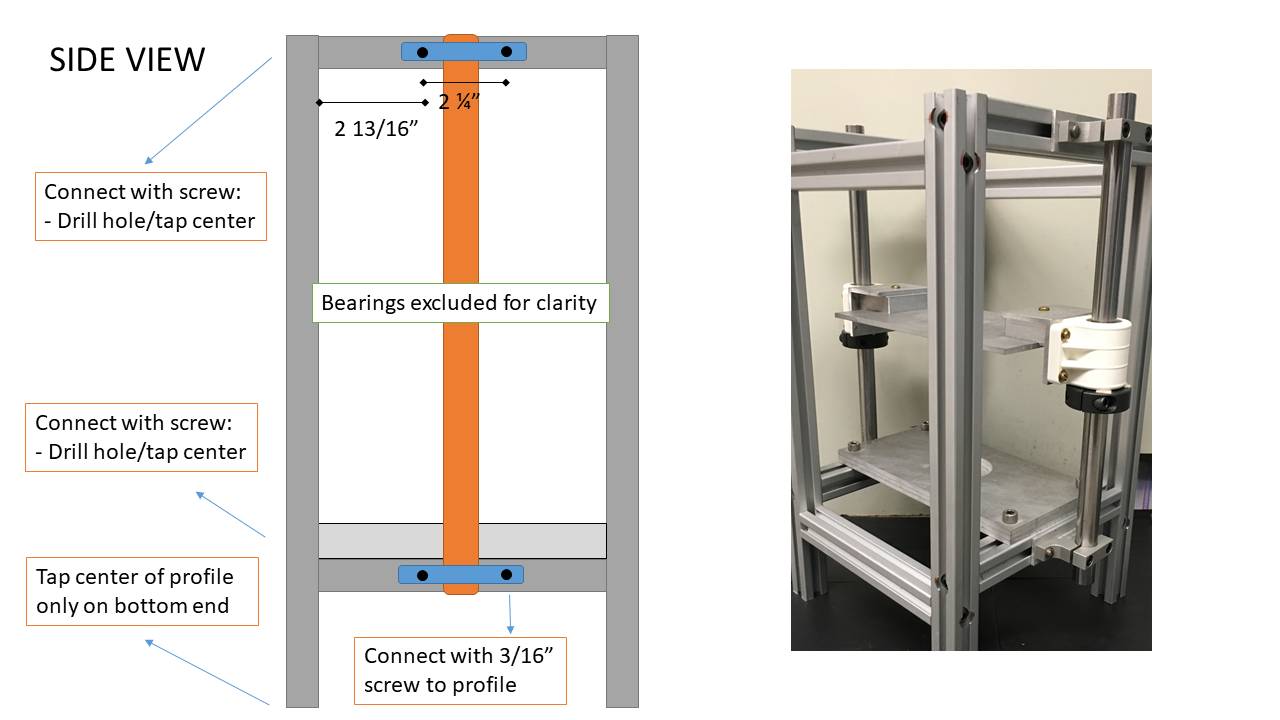


Figure S9: Assembled stand dimensions and pertinent notes. The shaft, shaft mounts, shaft clamp, and linear bearings were purchased from McMaster-Carr. The scheme for mounting the push plate to the bearings may be seen in the image. An L-shaped metal plate was mounted to the bearing and the plate was attached to this and centered along the bearing.

Figure S10: Additional image showing plate supports for mixer with design notes

Figure S11: Additional design details for the push plate attachment to the linear bearings on the shaft. As envisioned shown on left and as made shown at right. It is best to center the push plate vertically along length of the bearing housing.

1.
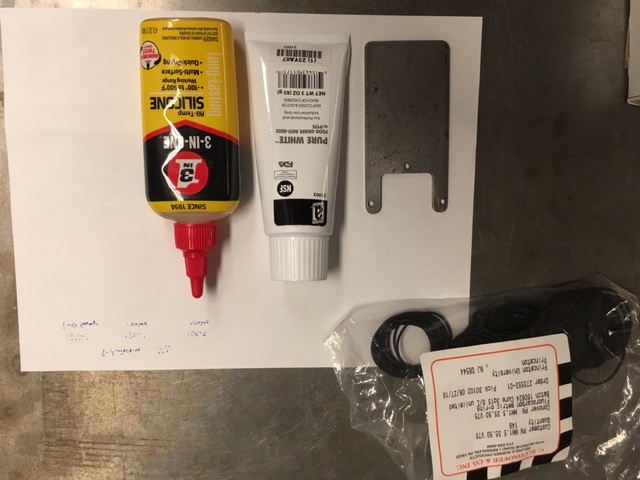
*Operating Notes*

**Replaceable Parts**

- Fluorocarbon O-ring (1.5mm, 35.50mm, V75 from Conover) – for sealing the mixing geometry. These are cheap and deteriorate over time – order a large batch to rotate through as needed.
- Luer fittings (1/4”-28 fitting, P-604 from IDEX): for attaching syringes to the top geometry
- VacuTight Fitting (1/4”-28 fitting, P-942x from IDEX): for outlet on the bottom fitting
- Outlet tubing for VacuTight fitting and cut to desired length

**Stand Design Parts**

- Linear Bearings (2): Linear ball bearing, fixed alignment. McMaster-Carr Part # 1052K13

Figure S12: Spanner wrench custom made for final tightening of the mixer.

- Shaft: 3/4” hardened steel (<30” needed). McMaster-Carr Part # 6061K545
- Shaft Mounts (4): quick access mounts. McMaster-Carr Part # 1865K5
- Shaft Stops (2): Extra-grip clamping two-piece shaft collar. McMaster-Carr Part # 8386K39
- ITEM Aluminum profiles: 1.5” diagonal, length as needed for design
- Aluminum plates for push plate and mixer support

**Additional Items Needed**

- Silicone lubricant- for bearings shaft on mixer stand
- Anti-sieze (we use a PTFE-based food grade option) – for periodic lubrication of the threading for the top and bottom pieces of the mixer
- A spanner wrench (custom-built, three pronged) – for tightening/loosening top piece of mixer. Have one fabricated to match the top metal piece.
- Gas-tight syringes with set screws attached to ensure equal heights (see Fig. S13) or single-sized plastic syringes (depending on syringe design, should accommodate up to 10 ml syringes).

**Maintenance and Cleaning**

1. About weekly, apply a few dabs of silicone lubricant to the two bearing shafts. Clean dust off.
2. As needed, apply a few dabs of anti-seize to the threading of the bottom metal piece. Spread evenly by screwing and unscrewing the top geometry a few times. If the pieces begin to catch during assembly, do not force them.
3. Clean the Delrin geometry after each run with an appropriate solvent. Do the same for the top geometry (where it contacts process fluid). Ensure that residual solvent is blown free from the syringe inlets with a little air or nitrogen.
4. About monthly, briefly polish the surface of the Delrin geometry with polishing paper and a little water (we use 15 micron silicon carbide).


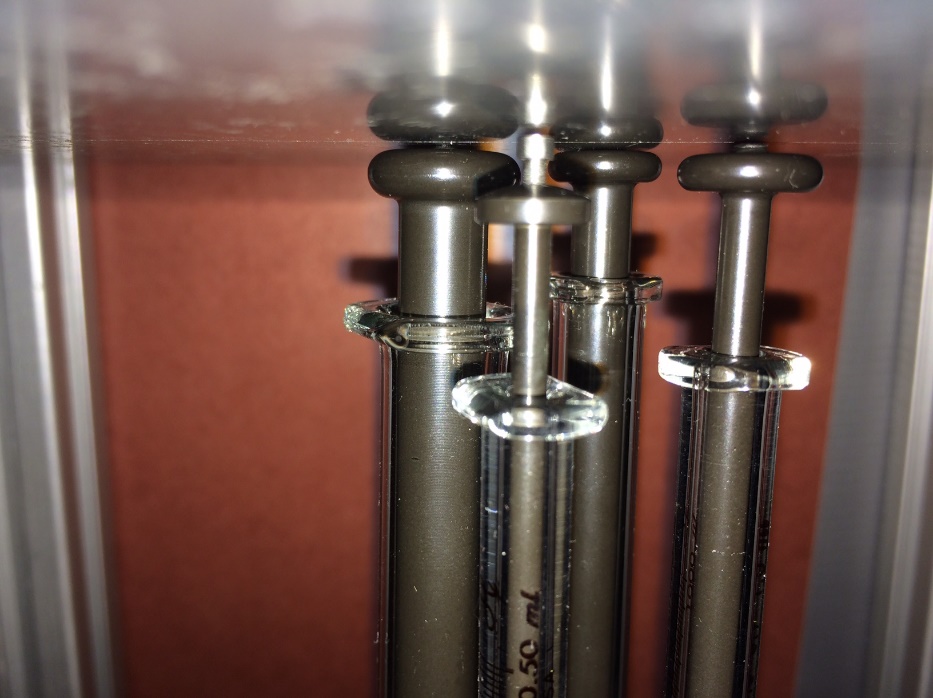

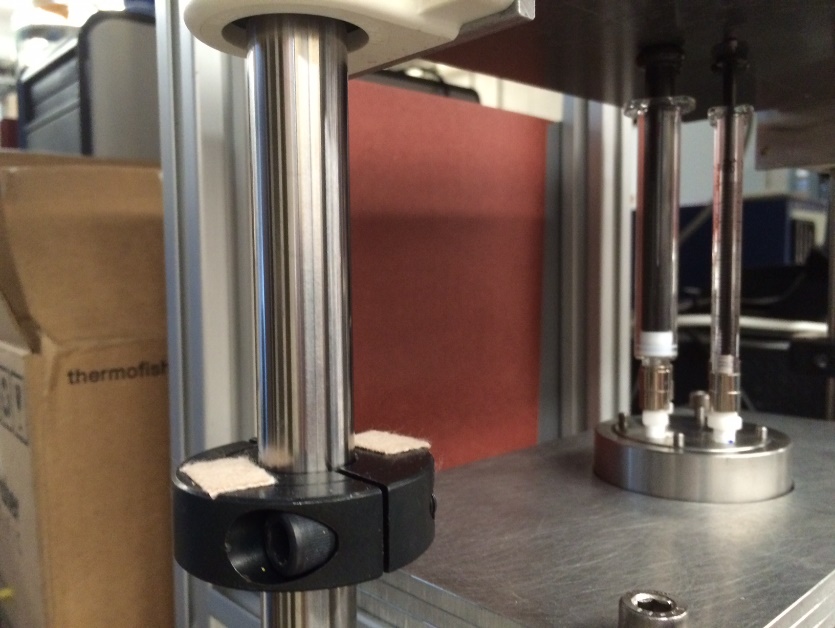


Figure S13: Syringe modification with set screws to ensure equal heights at the final travel distance when depressed with the pusher plate of the stand. The mechanical stops on the bearing shaft in the image on the right have been lowered for clarity.

**Basic Operation**

1. Place an O-ring in the Delrin geometry. Fit this snugly onto the top metal piece by aligning the pins, ensuring the O-ring does not come loose.
2. Loosen the outlet fitting on the bottom metal piece, then screw together the top and bottom components (along with the Delrin geometry) by hand. If the Delrin piece is able to slip off the dowels of the top piece, we recommend assembly “upside down” to avoid this.
3. Use the spanner wrench to tighten the fit to firmly snug.
4. Tighten the outlet fitting to form a seal with the Delrin geometry.
5. Place the mixer on the mixer stand and arrange a collection vial (e.g. a 15ml or 50 ml falcon centrifuge tube) underneath. Support the push plate to allow placement of the syringes unimpeded.
6. Prepare solvent and antisolvent solutions at the desired concentration and volume.
7. Using a syringe and a blunt tip needle, draw up each solution, remove air bubbles, and attach to the syringe fittings on the mixer. For best reproducibility, ensure you use a consistent order.
8. Double check syringe placement and that a collection vessel is present, then rapidly depress the syringes using the push plate.
9. Remove the collected solution and analyze/process as needed
10. Remove syringes from mixer over a waste container. This breaks surface tension and some solution will drip out. This should NOT be collected with the bulk. Hold-up is about 300 ul.
11. Take apart the mixer, clean the Delrin and top piece as noted above. You may need to use a fine flat head screwdriver to separate the Delrin geometry and the top metal piece. Be careful not to damage the Delrin surface.
12. *MIVM vs. μMIVM dimensions*

The dimensions listed below were used with the volumetric flow rate of each trial to calculate a linear velocity in the channel. This velocity was used with Eqn. 1 to calculate the Reynolds Number.

Table S1: Mixer dimension comparison between the original MIVM and the new μMIVM

|  | **Chamber Diameter** | **Channel Height** | **Channel Width** |
| --- | --- | --- | --- |
| μMIVM | 0.5 cm | 0.075 cm | 0.075 cm |
| MIVM | 0.6 cm | 0.15 cm | - 1. cm |

1. *Formulation Summaries*

The raw data from all mixer validation trials is summarized in the tables and figures included below to provide greater data clarity.

Table S2: Formulation summary for Reynolds Number dependence study. The “Syringe Depression Time” column has an uncertainty of 0.3 seconds. The inlet flow rate is for each stream rather than a total flow rate.

|  | **Syringe Depression  Time (s)** | **Inlet Flow Rate  (ml/min)** | **Re** | **Size (nm)** | **PDI** |
| --- | --- | --- | --- | --- | --- |
| **μMIVM (Stand-Driven) Summary** | | | | | |
| 1 | 0.8 | 75 | 65,795 | 88 | 0.20 |
| 2 | 0.6 | 100 | 87,727 | 68 | 0.25 |
| 3 | 0.6 | 100 | 87,727 | 70 | 0.23 |
| 4 | 6 | 10 | 8,773 | 134 | 0.17 |
| 5 | 7 | 9 | 7,519 | 146 | 0.17 |
| 6 | 8.5 | 7 | 6,192 | 158 | 0.20 |
| 7 | 3 | 20 | 17,545 | 99 | 0.15 |
| 8 | 3.5 | 17 | 15,039 | 109 | 0.17 |
| 9 | 4 | 15 | 13,159 | 118 | 0.16 |
| 10 | 0.6 | 100 | 87,727 | 73 | 0.23 |
| 11 | 1 | 60 | 52,636 | 83 | 0.18 |
| 12 | 1.5 | 40 | 35,091 | 94 | 0.18 |
| 13 | 1.4 | 43 | 37,597 | 93 | 0.16 |
| 14 | 0.6 | 100 | 87,727 | 71 | 0.22 |
| 15 | 6 | 10 | 8,773 | 157 | 0.16 |
| 16 | 0.5 | 120 | 105,272 | 87 | 0.20 |
| 17 | 2.5 | 24 | 21,054 | 108 | 0.14 |
| 18 | 0.4 | 150 | 131,590 | 85 | 0.22 |
| 19 | 0.7 | 86 | 75,195 | 78 | 0.21 |
| **MIVM Summary** | | | | | |
| 20 | NA | 1 | 359 | 275 | 0.23 |
| 21 | NA | 30 | 10,766 | 169 | 0.12 |
| 22 | NA | 5 | 1,794 | 225 | 0.27 |
| 23 | NA | 3 | 1,077 | 252 | 0.27 |
| 24 | NA | 60 | 21,533 | 140 | 0.09 |
| 25 | NA | 15 | 5,383 | 161 | 0.14 |
| 26 | NA | 8 | 2,871 | 174 | 0.13 |
| 27 | NA | 2 | 718 | 223 | 0.26 |
| 28 | NA | 12 | 4,307 | 172 | 0.13 |
| 29 | NA | 20 | 7,178 | 156 | 0.11 |
| 30 | NA | 68 | 24,404 | 130 | 0.07 |
| 31 | NA | 40 | 14,355 | 132 | 0.09 |
| 32 | NA | 25 | 8,972 | 146 | 0.11 |
| 33 | NA | 10 | 3,589 | 174 | 0.13 |
| **μMIVM (Pump-Driven) Summary** | | | | | |
| 34 | NA | 1 | 877 | 236 | 0.23 |
| 35 | NA | 30 | 26,318 | 106 | 0.09 |
| 36 | NA | 6 | 5,264 | 166 | 0.13 |
| 37 | NA | 4 | 3,509 | 214 | 0.30 |
| 38 | NA | 40 | 35,091 | 97 | 0.10 |
| 39 | NA | 15 | 13,159 | 124 | 0.12 |
| 40 | NA | 10 | 8,773 | 146 | 0.11 |
| 41 | NA | 2 | 1,754 | 216 | 0.22 |
| 42 | NA | 0.5 | 439 | 215 | 0.27 |
| 43 | NA | 8 | 7,019 | 156 | 0.11 |
| 44 | NA | 25 | 21,932 | 106 | 0.10 |

Table S3: Additional formulations summary

| **Sample** | **Size (nm)** | **PDI** |
| --- | --- | --- |
| Dextran Rep 1 | 80 | 0.23 |
| Dextran Rep 2 | 75 | 0.22 |
| Dextran Rep 3 | 80 | 0.28 |
| OVA | 116 | 0.17 |
| HRP | 167 | 0.16 |
| Adjacent/Combined Rep 1 | 65 | 0.23 |
| Adjacent/Combined Rep 2 | 64 | 0.23 |
| Adjacent/Combined Rep 3 | 55 | 0.24 |
| Opposite/Combined Rep 1 | 59 | 0.21 |
| Opposite/Combined Rep 2 | 51 | 0.24 |
| Opposite/Combined Rep 3 | 58 | 0.20 |
| Opposite/Separate Rep 1 | 96 | 0.20 |
| Opposite/Separate Rep 2 | 89 | 0.18 |
| Opposite/Separate Rep 3 | 87 | 0.16 |

Figure S14: The Mixer Re dependence of NP size (PS/PS-PEG particles) using the MIVM or the μMIVM under either pump- or stand-driven flow

Table S4: Biologic formulations produced by iFNP in the μMIVM. The syringe orientation is shown schematically in Fig. 3A. Solutions were molecularly dissolved and mixed thoroughly before loading in the syringe.

| Formulation | Dextran | OVA | HRP |
| --- | --- | --- | --- |
| Syringe 1 | 0.2 ml  90/10 DMSO/Water  5 mg/ml Dextran  5 mg/ml PS-*b*-PAA | 0.5 ml  90/10 DMSO/Water  5 mg/ml OVA | 0.2 ml  90/10 THF/Water  1 mg/ml HRP  2 mg/ml PS-*b*-PAA |
| Syringe 2 | 1 ml chloroform | 0.5 ml THF  5 mg/ml PS-*b*-PAA | 0.9 ml chloroform  0.1 ml methanol  0.4 mg/ml Zn(NO_3_)_2_ |
| Syringe 3 | 1 ml chloroform | 0.5 ml chloroform | 1 ml chloroform |
| Syringe 4 | 1ml chloroform | 0.45 ml chloroform  0.05 ml methanol  6.7mg/ml Zn(NO_3_)_2_ | 1 ml chloroform |
| Collection Diluent | - | 4 ml chloroform | - |

Table S5: The effect of stream configuration on NP size in the μMIVM. NPs with a PS core and stabilized by PS-b-PEG were produced in triplicate at Re 90,000. SD is the standard deviation of the samples.

| Stream Orientation | Component Location | NP Diameter (nm) | | SD (nm) |
| --- | --- | --- | --- | --- |
| Adjacent | Combined | 61 | 5 | |
|  | Separated | 70 | 2 | |
| Opposite | Combined | 56 | 4 | |
|  | Separated | 91 | 5 | |

1. *PDI dependence on mixing rate*

The PDI is an indication of width or span of the size distribution of nanoparticles. Generally, a low polydispersity sample is desired (around a value of 0.15). The figures below depict the PDI as a function of Reynolds Number and mixing chamber residence time. This was calculated using the total volumetric flow rate and the chamber volume. There is additional time between the chamber and the point when the stream contacts the collection tube solution (i.e. water) that extends the total “incubation” time by about 10 fold. The outlet tubing ID was 0.15 cm.

Figure S15: PDI dependence on Reynolds Number of NPs produced using the MIVM and μMIVM. NPs were PS/PS-b-PEG. These data show good agreement between the two mixer designs under identical production conditions. High Re cannot be achieved with pump-driven flows due to mechanical constraints.





Figure S16: NP PDI dependence of the μMIVM on chamber residence time, calculated from the total volumetric flow rate and the chamber volume. NPs were PS/PS-b-PEG.

The PDI was not lowered by allowing longer incubation or assembly time after exiting the mixing chamber, as seen in Table S6. This indicates that high shear in the outlet tubing leads to aggregation. The transition to increasing PDI is observed when the residence time falls below the assembly time and the outlet flow becomes turbulent, as seen in Fig. S17.

Table S6: The effect of modifying outlet tube length to increase "incubation" time before reaching the quench. "Res. Time" is the total time (chamber + outlet tubing) before the particle solution reaches the quench bath.

| Tube Length | Res. Time | PDI (avg) | Replicate | PDI |
| --- | --- | --- | --- | --- |
| short | 19 ms | 0.24 | 1 | 0.27 |
|  |  |  | 2 | 0.24 |
|  |  |  | 3 | 0.22 |
| long | 110 ms | 0.23 | 1 | 0.27 |
|  |  |  | 2 | 0.17 |
|  |  |  | 3 | 0.26 |

Figure S17: PDI of formulations produced in the μMIVM as a function of the Reynolds Number in the outlet tube. The increase in PDI becomes noticeable as the tube transitions to turbulent flow (above Re~2000 ).

While the trend is clear, the impact on the quality of particles produced in the μMIVM is minimal. Fig. S18 shows the correlation function measured by the DLS at two very different Re values. No gross changes are observable.

Figure S18: DLS correlation functions for particles prepared at Reynolds Values of 37,000 or 132,000 showing minimal differences in curve shape. The values have been rescaled to overlay in order to facilitate comparison.
